# Physician's manual reporting underestimates mortality: evidence from a population-based HIV/AIDS treatment program

**DOI:** 10.1186/1471-2458-10-642

**Published:** 2010-10-25

**Authors:** Christopher G Au-Yeung, Aranka Anema, Keith Chan, Benita Yip, Julio SG Montaner, Robert S Hogg

**Affiliations:** 1 British Columbia Centre for Excellence in HIV/AIDS, Vancouver, British Columbia, Canada; 2 Faculty of Medicine, University of British Columbia, Vancouver, British Columbia, Canada; 3 Faculty of Health Sciences, Simon Fraser University, Burnaby, British Columbia, Canada

## Abstract

**Background:**

In clinical and cohort research, mortality estimates are often derived from manual reports generated by physicians or electronic reports from vital event registries. We examined the rate of underreporting of deaths by manual methods as compared with electronic reports from a vital event registry.

**Methods:**

The retrospective analyses included deaths among participants registered in an observational cohort who initiated highly-active antiretroviral therapy (HAART) between August 1, 1996 and June 30, 2006. Deaths were routinely reported manually by physicians and through annual electronic record linkages with a population-based vital event registry. Multivariate logistic regression was carried out to assess independent predictors of death reporting by manual methods.

**Results:**

Of the 3,116 individuals included in the analyses, 622 (20.0%) died during follow-up. Manual reporting by physicians only identified 377 (60.6%), while electronic linkages captured 598 (96.1%) of all deaths. Multivariate analysis indicated that deaths among individuals with lower CD4 cell count, higher HIV plasma viral load, a history of injection drug use, and under the care of an HIV-experienced physicians were more likely to be reported manually. Furthermore, non-accidental deaths were more likely to be reported manually, and manual reporting of deaths increased over time.

**Conclusions:**

Relying only on manual reports to ascertain deaths significantly underestimates the total number of deaths in the population. This can generate important biases when evaluating the impact of therapeutic interventions in the populational setting.

## Background

Accurate estimates of mortality are necessary for HIV surveillance, including assessments of antiretroviral treatment programs [1-4]. Several methods can be used to record deaths in a population, including physician reporting, vital statistics, and hospital registries [1,2]. Sampling-based approaches, verbal autopsies, morgue and burial data provide additional proxies, particularly in settings where vital event data may not be available [5-9].

Reporting methods for HIV deaths vary in terms of their sensitivity and specificity [10-13]. Autopsy and chart reviews, for example, have better specificity than death certificates since they describe the underlying causes of death in detail [3,4]. In clinical and cohort research, mortality estimates are often derived from manual reports generated by physicians [10] or electronic reports from vital event registries [14,15]. Previous studies of the HIV-positive and HIV-negative population have found varying degrees of agreement between deaths reported manually by physicians and population census data [16-20].

In this analysis, data derived from a population-based cohort of HIV-infected individuals on HAART in British Columbia (BC), Canada, are used to compare the number of deaths reported manually by physicians to the number of deaths reported electronically by a vital event registry.

## Methods

This study is based on HIV-positive men and women at least 18 years of age in the HAART Observational Medical Evaluation and Research (HOMER) cohort. HOMER is a prospective, observation population-based cohort of individuals enrolled in a province-wide HIV/AIDS Drug Treatment Program (DTP) in BC, Canada. The HOMER cohort includes data on individuals who entered the DTP antiretroviral-naïve to HAART between August 1, 1996 and June 30, 2006, with follow-up to June 30, 2007.

The HIV/AIDS DTP and the HOMER cohort have been approved by the University of British Columbia (UBC) Research Ethics Board at its St Paul's Hospital site. Individuals in HOMER were not required to provide written informed consent for the purposes of the analyses presented herein. These administrative analyses occur within the context of a universal health care system in which individuals receive medical care, laboratory monitoring, and HAART free of charge.

If a death occurs among an individual enrolled in the DTP, the HAART prescribing and/or primary care physician is responsible to report the death to the program (manual reporting). A record linkage between the DTP and the BC Vital Statistics death registry is also performed annually to capture all deaths among individuals enrolled in the DTP (electronic reporting). In addition to these death data, the HOMER cohort captures clinical indicators and socio-demographic information among individuals enrolled in the DTP.

Analyses included bivariate comparisons of individuals who died and did not die and multivariate logistic regression to examine factors associated with manual methods of reporting deaths among the deceased. The following covariates were considered in these analyses: physician practice setting (dichotomized into rural and urban categories according to Statistics Canada Census denominations [21,22]); HIV experience among physicians (defined as the number of HIV-positive individuals the physician had previously treated at the time a patient was enrolled into the DTP [5]); calendar year of death, cause of death, age, sex, history of injection drug use, adherence to HAART (defined as the number of days of antiretroviral medications dispensed divided by the number of days of follow-up during first year of treatment, and expressed as a percent [23]), and baseline CD4 cell count and HIV plasma viral load. CD4 cell count was recorded using flow cytometry and fluorescent monoclonal antibody analysis (Beckman Coulter, Inc., Mississauga, Ontario, Canada), and HIV viral load was measured using the Roche Amplicor Monitor assay (Roche Diagnostics, Laval, Quebec, Canada) using either the standard method or the ultrasensitive adaptation.

All analyses are conducted using SAS version 9.1.3 (SAS, Cary, North Carolina, United States). Statistical tests are two-sided and p-values of less than 0.05 are considered statistically significant.

### Ethical Statement

Ethical approval was requested and obtained for this study. The HIV/AIDS Drug Treament Program and the HOMER cohort have been approved by the University of British Columbia (UBC) Research Ethics Board at its St Paul's Hospital site.

## Results

Between August 1, 1996 and June 30, 2006, 622 (20.0%) deaths occurred among the 3,116 individuals in HOMER. Manual reporting identified 377 (60.6%), while electronic linkages captured 598 (96.1%) of all deaths. Manual reporting identified 377 (60.6%), while electronic linkages captured 598 (96.1%) of all deaths. Manual reporting alone captured only 24 (3.9%) deaths, while vital statistics alone captured 245 (39.4%) of all deaths. The remaining 353 (56.8%) deaths were reported by both sources. Based on a review of the principal cause of death, the vast majority of deaths (535 [86%]) were deemed non-accidental (i.e. HIV-related (373 [60%]), circulatory system-related (35 [5.6%]), malignancy-related (21 [3.4%]), hepatitis-related (19 [3%]), respiratory-system related (15 [2.4%]), digestive system related (14 [2.2%]), according to the *International Statistical Classification of Diseases and Related Health Problems *coding system [24]. The remaining 87 (14.0%) deaths were deemed accidental (i.e. accidental poisoning by and exposure to noxious substances (60 [9.6%]), intentional self-harm (16 [2.6%]).

In bivariate analyses, deaths were more likely to occur among individuals who were older (median age: 41 *versus *39; p < 0.001), had a history of injection drug use (37.3% *versus *27.4%; p < 0.001), had a less HIV-experienced physician (median number of HIV-positive patients: 30 *versus *70; p < 0.001), had an AIDS defining illness at baseline (17.7% *versus *14.3%; p = 0.038), had a lower CD4 cell count at baseline (median CD4: 140 cells/mm^3 ^*versus *200 cells/mm^3^; p < 0.001), had a plasma HIV viral load ≥100,000 copies/mL at baseline (65.9% *versus *53.0%; p < 0.001), and had lower adherence to HAART (61.7% *versus *39.2% of patients who are < 95% adherent to HAART; p < 0.001).

Table 1 shows the results of the multivariate logistic regression examining factors associated with manual methods of reporting deaths among the deceased. Deaths among those with lower CD4 cell count, higher HIV plasma viral load, a history of injection drug use, and under the care of an HIV-experienced physician were more likely to be reported manually. Furthermore, non-accidental deaths were more likely to be reported manually, and manual reporting of deaths increased over time. Of the 245 patients captured by vital statistics, 16 (6.5%) were lost to follow-up (not seen over 6 months) versus 4(1.1%) of the 377 patients captured by manual reporting. Using a lost to follow-up definition of "not seen in over 12 months", vital statistics recorded 6 (2.4%) patients versus 1(0.3%) by manual reporting. The number of deaths captured by vital statistics compared to manual reporting was still significantly different after accounting for loss to follow-up (p < 0.05).

**Table 1:** Unadjusted and adjusted models showing independent predictors of manual reporting of deaths

Variable		Unadjusted	Adjusted
		OR (95% CI)	OR (95% CI)
**Physician practice location**	Urban *versus *rural	0.79 (0.47 to 1.32)	--
**Physician HIV-experience**	*per *10 patient increase	1.06 (1.01 to 1.11)	1.02 (1.00 to 1.04)
**Year of death**	*per *1 year increase	1.18 (1.11 to 1.26)	1.17 (1.09 to 1.25)
**Cause of death**	Non-accidental *versus *Accidental	2.23 (1.41 to 3.53)	1.69 (1.03 to 2.78)
**Age**	*per *10 year increase	0.99 (0.85 to 1.17)	--
**Sex**	Female *versus *Male	0.84 (0.56 to 1.27)	--
**History of injection drug use**	Yes *versus *No	1.57 (1.12 to 2.20)	1.49 (1.03 to 2.15)
**Baseline CD4 cell count (Cells/mm^3^)**	*per *100 cells/mm^3^ increase	0.88 (0.81 to 0.95)	0.90 (0.83 to 0.98)
**Baseline plasma viral load** **(Log_10 _copies/mL)**	*per *Log_10 _copies/mL increase	1.29 (0.99 to 1.69)	1.38 (1.04 to 1.85)
**Adherence**	≥ 95% *versus *< 95%	1.44 (1.03 to 2.02)	1.41 (0.99 to 2.01)

OR, odds ratio; CI, confidence interval.

## Discussion

Our results demonstrate that manual methods of death reporting by physicians underestimated the total number of deaths in the population by 40%. Annual electronic linkages with the vital statistics death registry captured 96% of deaths, suggesting an improved death registry sensitivity. The remaining 4% of deaths not identified through this linkage may be attributed to missing data or discrepancies in the patient identifiers being matched. While electronic linkages identify a high proportion of the mortality cases, the disadvantage is that there must be accurate, up-to-date demographic information such as names, birthdates, and health care numbers for the matching process. Even though manual reporting only covers about 60% of all deaths in our cohort, any patient identifier discrepancies can be easily clarified with the physician office staff members.

Our findings are similar to those identified by the United States Centres for Disease Control, which found that only 54% of deaths had been manually reported to the District of Columbia between 2000 and 2005 [17]. In this study, the higher proportion of deaths captured by electronic methods as compared to manual methods suggests that electronic record linkage is essential to accurately ascertain deaths among persons with HIV [17]. The improved rate of manual reporting in our study could be a consequence of its longer duration (1996-2006) and enhanced physician training for manual reporting over time. Improvements in reporting can enhance the accuracy of HIV prevalence estimates and distribution of HIV treatment and prevention resources in regions with the highest burden [17].

Studies from the United States suggest that underreporting may be due to lack of physician knowledge about the administrative process for reporting deaths, and intentional non-reporting to protect patient confidentiality [16]. It is promising to note that manually reporting of deaths has increased over time in our cohort. Also of interest, HIV-experienced physicians were more likely to manually report deaths in our study. This suggests that educational initiatives may be able to enhance manual death reporting when vital registration systems are not available or accurate, such as in resource-limited settings. Additional physician or health working training for those caring for HIV-infected individuals may be required to effectively quantify the actual number of deaths.

Access to vital registration records could be an invaluable resource to effectively ascertain the number of deaths among patients enrolled in a population based HIV/AIDS treatment program. Cohort studies relying solely on manually reported data may be significantly underestimating actual mortality. In addition, patients recorded as lost to follow up may not be captured as a death by vital registration systems. Thus, this data limitation calls for studies to analyze separate outcomes based on the time to loss to follow up and the time to death.

Our study has some strengths and limitations. Strengths include having a large observational database and using physician reported data in conjunction with vital statistics records to obtain socio-demographic and clinical information on patients. However, the HOMER cohort provides varying amounts of detail on each patient's clinical and socio-demographic characteristics. Specifically, patient ethnicity was unknown for more than half of the 622 deaths. Therefore, we were unable to compare differences between the number of HIV deaths reported by physicians and the death registry stratified by patient ethnicity. Inclusion of this variable would have been relevant, given that studies in the United States have found disagreement between physician and vital statistic reporting of deaths by ethnic group [18-20]. We also realize that confounder variables such as socio-economic status may have influenced our results, however, this classification is not made for individuals in the HOMER cohort.

## Conclusion

Our results demonstrate that physicians' manual HIV death reports underestimated mortality among individuals on HAART in BC by 40%. Cohorts relying only on physician reported deaths are potentially underreporting the total number of deaths among HIV-infected individuals. This represents a major limitation for studies using exclusively physicians' manual HIV death reports to ascertain mortality rates, as this practice can generate important biases when evaluating the impact of therapeutic interventions in the populational setting. The use of both manually reported data systematically cross-checked and supplemented with vital registration records is ideal for capturing the majority of deaths in an observational cohort of HIV-infected patients.

## Competing interests

RS Hogg has held grant funding from the National Institutes of Health, Canadian Institutes of Health Research National Health Research Development Program, and Health Canada. He has also received funding from Agouron Pharmaceuticals Inc, Boehringer Ingelheim Pharmaceuticals Inc, Bristol-Myers Squibb, GlaxoSmithKline, and Merck Frosst Laboratories for participating in continued medical education programmes. JSG Montaner has received grants from, served as an ad hoc advisor to, or spoken at various events sponsored by Abbott, Argos Therapeutics, Bioject Inc, Boehringer Ingelheim, BMS, Gilead Sciences, GlaxoSmithKline, Hoffmann-La Roche, Janssen-Ortho, Merck Frosst, Pfizer, Schering, Serono Inc, TheraTechnologies, Tibotec, Trimeris. He has also held grant funding from the Canadian Institutes of Health Research and National Institutes of Health. He has also received funding for research and continuing medical education programs from a number of pharmaceutical companies including Abbott, Boehringer Ingelheim, and GlaxoSmithKline.

## Authors' contributions

CGA, AA, KC, BY, JSGM and RSH contributed to the study design, data gathering, interpretation of results and manuscript draft. KC performed the analysis. CGA edited the manuscript. All authors revised the manuscript critically for important intellectual content; and gave final approval of the version to be submitted.

## Pre-publication history

The pre-publication history for this paper can be accessed here:

http://www.biomedcentral.com/1471-2458/10/642/prepub
